# Applications of *Lactobacillus acidophilus*-Fermented Mango Protected *Clostridioides difficile* Infection and Developed as an Innovative Probiotic Jam

**DOI:** 10.3390/foods10071631

**Published:** 2021-07-14

**Authors:** Bao-Hong Lee, Wei-Hsuan Hsu, Hao-Yuan Chien, Chih-Yao Hou, Ya-Ting Hsu, You-Zuo Chen, She-Ching Wu

**Affiliations:** 1 Department of Horticulture, National Chiayi University, Chiayi 600355, Taiwan; bhlee@mail.ncyu.edu.tw (B.-H.L.); s1072093@mail.ncyu.edu.tw (H.-Y.C.); 2 Department of Food Safety/Hygiene and Risk Management, College of Medicine, National Cheng Kung University, Tainan 701401, Taiwan; whhsu@mail.ncku.edu.tw (W.-H.H.); sc6094017@gs.ncku.edu.tw (Y.-T.H.); xzp666842@gmail.com (Y.-Z.C.); 3 Center of Allergy and Mucosal Immunity Advancement at the National Cheng Kung University, Tainan 701401, Taiwan; 4 Department of Seafood Science, National Kaohsiung University of Science and Technology, Kaohsiung 81157, Taiwan; chihyaohou@gmail.com; 5 Department of Food Science, National Chiayi University, No. 300 Syuefu Rd., Chiayi 600355, Taiwan

**Keywords:** jam, *Lactobacillus acidophilus*, *Clostridioides difficile*, mango, *Faecalibacterium prausnitzii*

## Abstract

*Clostridioides difficile* infection (CDI) is a large intestine disease caused by toxins produced by the spore-forming bacterium *C. difficile*, which belongs to Gram-positive bacillus. Using antibiotics treatment disturbances in the gut microbiota and toxins produced by *C. difficile* disrupt the intestinal barrier. Some evidence indicates fecal microbiota transplantation and probiotics may decrease the risk of CDI recurrence. This study aimed to evaluate the efficacy of fermented mango by using the lactic acid bacteria *Lactobacillus acidophilus* and develop innovative products in the form of fermented mango jam. *L. acidophilus*-fermented mango products inhibited the growth of *C. difficile* while promoting the growth of next-generation probiotic *Faecalibacterium prausnitzii*. Both supernatant and precipitate of mango-fermented products prevented cell death in gut enterocyte-like Caco-2 cells against *C. difficile* infection. Mango-fermented products also protected gut barrier function by elevating the expression of tight junction proteins. Moreover, *L. acidophilus*-fermented mango jam with high hydrostatic pressure treatment had favorable textural characteristics and sensory quality.

## 1. Introduction

The incidence of *Clostridioides difficile* (formerly *Clostridium difficile*) infection (CDI) is 10-fold higher in older adults than in young adults [1]. A clinical study found that the abundance of several next-generation probiotics, including *Akkermansia muciniphila*, *Escherichia coli*, and *Klebsiella* spp., was higher in patients with CDI than in controls, suggesting these bacterial populations may be involved in CDI development [2]. However, a reduction in the number of butyrate-producing bacteria, such as *Lachnospira* spp., *Butyricimonas* spp., and *Faecalibacterium prausnitzii*, was found in patients with CDI [3,4]. Bacterium *F. prausnitzii* is Gram-positive, nonspore-forming, and nonmotile [5]. *F. prausnitzii* represents about 5% of the total population of intestinal bacteria, which is one of the most abundant species in the gut microbiota of healthy adults and is the predominant butyrate-producing bacterium in the gastrointestinal tract. The d-lactate and butyrate (>10 mM butyrate) were major end products of *F. prausnitzii* during glucose fermentation [6,7]. *F. prausnitzii* contributes to the establishment of epithelial homeostasis, which modulates colonic goblet cells and gut barrier function [8]. Moreover, *F. prausnitzii* and its cultural supernatant alleviate intestinal inflammation in the colitis mice model by reducing intestinal permeability [9]. The mechanism underlying the protective effects of *F. prausnitzii* mediated by elevating tight junction proteins [10]. A monolayer of epithelial cells forms an interface between the host and the microbiota that colonizes the gastrointestinal tract. Commensal gut microbiota and their metabolites interact with epithelial cells in the intestine of humans. A study reported that *F. prausnitzii*-derived extracellular vesicles have the potential for promoting activation of Toll-like receptors and intestinal immunity [11], these indicate that *F. prausnitzii* is essential to health of hosts. There is increasing evidence that metabolites from *F. prausnitzii* regulate the intestinal epithelium. Medium supplemented with flavin and cysteine or glutathione has been shown to support the growth of *F. prausnitzii* [12]. In addition, *F. prausnitzii* uses mucin (MUC) as its sole energy source and is considered one of the “next-generation probiotics.”

Numerous lactic acid bacteria and yeasts have been reported to use mango as nutrients thereby producing postbiotics [13,14,15,16,17]. Studies have demonstrated that the polyphenolic compounds of mango peel and mangiferin conversion were increased through gastrointestinal digestion and gut microbiota fermentation [18,19]. Moreover, mango peel aids in the regulation of gut microbiota, including an increase in the abundance of *Faecalibacterium*, *Bifidobacterium*, *Roseburia*, *Eubacterium*, *Catenibacterium*, *Prevotella*, *Phascolarctobacterium*, *Collinsella*, and *Bacteroides* genera [20].

MUC expression has been usually determined during the growth and differentiation of the enterocyte-like Caco-2 and goblet cell-like LS174T cells. Caco-2 (expressing MUC-1, MUC-3, MUC-4, MUC-5A/C, and MUC-13) and LS174T (expressing MUC-1, MUC-2, and MUC-6) cell lines have been discovered great in vitro models for research the specific mechanisms responsible for expressing mucin [21]. It has been reported that oligosaccharides including stachyose, cellobiose, raffinose, lactulose, and chitooligosaccharides can affect the adhesion of *F. prausnitzii* to mucus-secreting intestinal epithelial cells [22]. Therefore, we investigated the effects of *Lactobacillus acidophilus* BCRC14079-fermented mango on the growth of *F. prausnitzii* and the protection of Caco-2 cells for the intestinal biological role in human health. Finally, the preparation conditions of *L. acidophilus* BCRC14079-fermented mango jam was investigated.

## 2. Materials and Methods

### 2.1. Chemicals

Fetal bovine serum (FBS) was purchased from Life Technologies (Auckland, New Zealand). Dimethyl sulfoxide (DMSO) was obtained from Wako Pure Chemical Industries (Saitama, Japan). Triton X-100, trypsin, and sodium dodecyl sulfate (SDS) were purchased from Sigma Chemical Co. (St Louis, MO, USA). Dulbecco’s modified Eagle’s medium (DMEM), streptomycin, and penicillin were purchased from HyClone Laboratories (Logan, UT, USA). Lactobacilli MRS broth was purchased from Difco Laboratories (Detroit, MI, USA). BHI medium was obtained from Thermo Fisher Scientific (Waltham, MA, USA).

### 2.2. Fermentation

The pulp of mango fruit was homogenized into a puree using a homogenizer, freeze-dried to a powder, and stored at −80 °C. The mango powder was formulated (30%) as a culture medium and fermented with *L. acidophilus* (BCRC14079; Food Industry Research and Development Institute, Hsin Chu, Taiwan) for 1, 3, and 5 d. The bacterial counts, pH level, and titratable acidity were determined on days 1, 3, and 5. pH value was detected by a digital pH meter (OHAUS Corporation, Parsippany, NJ, USA) that calibrated with pH 4.0 and 7.0 buffers. In titratable acids measurements, 5 mL of the tested sample was used and titrated by 0.1 N sodium hydroxide (NaOH) to pH 8.2.

*L. acidophilus* BCRC14079 was cultured in MRS broth under anaerobic conditions at 37 °C by using an atmosphere generation system (Oxoid, Basingstoke, UK). The number of the lactobacilli was detected on MRS agar plates under anaerobic cultivation.

The fermented products were centrifuged and divided into the *L. acidophilus* BCRC14079-fermented mango supernatant and *L. acidophilus* BCRC14079-fermented mango precipitate. Water extracts of unfermented mango were used as controls. *L. acidophilus* BCRC14079-fermented mango supernatant was filtered through a 0.22 μm filter. *L. acidophilus* BCRC14079-fermented mango precipitate contained the biomass of bacteria. Therefore, it was extracted with 100 °C water for 1 h and the solution was then filtered through a 0.22 μm filter. Finally, these products (*L. acidophilus* BCRC14079-fermented mango supernatant and *L. acidophilus* BCRC14079-fermented mango precipitate–water extract) were freeze-dried and then stored at −80 °C until use in the intestinal cell and microbial experiments.

### 2.3. Cell Culture

Cell culture and treatment Caco-2 cell line was purchased from the Bioresource Collection and Research Center (BCRC, Food Industry Research and Development Institute, Hsin Chu, Taiwan). Cells were grown in a DMEM medium that contained 10% heat-inactivated FBS, 2 mM of L-glutamine, and 2 mM of glutamine in a humidified atmosphere of 95% air and 5% CO_2_ under 37 °C cultivation.

### 2.4. Western Blotting

An ice-cold buffer containing 1% of Triton X-100, 0.1% of SDS, 500 mM of sodium vanadate, 20 mM of Tris-HCl (pH 7.4), 10 mM of NaF, 2 mM of EDTA, 1 mM of phenyl-methanesulfonyl fluoride, and 10 mg/mL of aprotinin was used to lyse the cells. The supernatant of cells was obtained from centrifuged (12,000× *g*, 10 min) cell lysate. SDS–PAGE (10%) was used to resolve the proteins and transferred them to a polyvinylidene fluoride membrane. Nonfat milk (5%) was used to block membranes for 1 h, and then primary antibodies were added to membranes for 2–4 h. Subsequently, the membrane was washed with phosphate-buffered saline with Tween-20 (PBST) for 5 min three times and incubated with horseradish peroxidase (HRP)-linked secondary antibody for 1 h. After washing three times with PBST, the enhanced chemiluminescent reagent (Millipore, Billerica, MA, USA) was used to determine the protein concentration.

### 2.5. L. acidophilus BCRC14079-Fermented Mango Inhibited C. difficile Growth

The *C. difficile* 630 strain (ATCC^®®^ BAA-1382™) was anaerobically cultured in brain heart infusion (BHI) medium with 0.05% l-cysteine for 16 h at 37 °C and then refreshed the broth until grown to early stationary phase (OD_600_ ≈ 0.7). Subsequently, *C. difficile* culture (1 × 10^6^ CFU/mL) was added into fresh BHI broth containing 31.25, 62.5, 125, 250, and 500 μg/mL *L. acidophilus* BCRC14079-fermented mango samples. Three independent samples were analyzed for each experiment. OD_600_ was measured after culturing the cells for different times.

### 2.6. C. difficile Infection in Caco-2 Cells

Caco-2 cells were incubated with DMEM medium without antibiotics and FBS before being infected. After overnight incubation, a pellet of *C. difficile* was collected and resuspended in anaerobic presterilized DMEM. The bacterial suspension was used to infect Caco-2 cells with the infected ratio of 100:1 (bacteria:cell) anaerobically for 30–180 min. Cell viability was determined by using the reduced mitochondrial activity (MTT) assay according to the manual (Sigma-Aldrich Chemical Co., St Louis, MO, USA).

### 2.7. Investigation of L. acidophilus BCRC14079-Fermented Mango Regulated Growth of F. prausnitzii

*F. prausnitzii* (BCRC81047) was obtained from Food Industry Research and Development Institute, Hsin Chu, Taiwan, which was cultured in YCFA medium in anaerobic condition for 24 h at 37 °C (YCFA medium is formulated according to American Type Culture Collection recommendations). After overnight incubation, bacterial pellets were then refreshed in YCFA broth until to grow to early stationary phase (OD_600_ ≈ 0.8). Subsequently, fresh *F. prausnitzii* (1 × 10^6^ CFU/mL) was then added to YCFA broth, which contained 125, 250, and 500 μg/mL of samples. Triplicate repeats were analyzed for each experiment. OD_600_ was measured after culturing the cells for different times.

### 2.8. Assay for Short-Chain Fatty Acids (SCFAs)

The bacteria cultural solution was collected and centrifuged at 13,000× *g* and 4 °C for 15 min to obtain the supernatant for subsequent analysis. The levels of SCFAs such as acetic acid, propionic acid, and butyric acid were performed by gas chromatography–flame ionization detection (GC–FID) that used the Shimadzu GC-2010 (Shimadzu Corp, Tokyo, Japan) with a capillary column (BP21 FFAP 30 m × 0.53 mm i.d., 0.50 µm film thickness, Trajan, Melbourne, Australia). The carrier gas was nitrogen, and the splitless injection volume was 1 µL. Auxiliary gases for the flame ionization detector were hydrogen (30 mL/min of flow rate) and dry air (300 mL/min of flow rate). The temperatures of the injector and detector were 220 °C and 240 °C, respectively. The temperature of the GC oven was first set at 90 °C for 1 min and elevated to 150 °C at 10 °C/min, and then to 200 °C at 20 °C/min and following held for 1 min. Triplicate repeats were analyzed, and the obtained data were normalized to the concentrations of external standards and are showed in μM [23].

### 2.9. Preparation of L. acidophilus BCRC14079-Fermented Mango Jam Treated with High-Pressure Processing (HPP)

The *L. acidophilus* BCRC14079-fermented mango solution was mixed thoroughly with sucrose and pectin. The pH was maintained at 3.05 using citric acid. The pulp solution was divided into HPP-treatment and heat-treatment groups. For HPP treatment, *L. acidophilus* BCRC14079-fermented mango pulp was processed at 150, 300, and 500 MPa after being vacuum-packaged in a plastic bag. The processing time and temperature were 20 min and 25 °C, respectively [24]. For heat treatment, *L. acidophilus* BCRC14079-fermented mango pulp was mixed with sucrose and pectin and heated by a gas burner at 180 °C. Total soluble solids were monitored during boiling. The solution was continuously stirred and stopped heating when the soluble solids were reached at 65° Brix. The mixture was then poured into glass beakers to cool under ambient conditions.

### 2.10. Texture Profile Analysis (TPA)

The texture profile analysis (TPA) assay of *L. acidophilus* BCRC14079-fermented mango jam (60 g; sample height was 24 cm) was performed by a texture analyzer (RapidTA^®®^ Texture analyzer, Horn Instruments Co., Ltd., Taoyuan, Taiwan). A knife blade probe (RP40) was used, and the trigger was compressed to 40% at a 1 mm/s speed rate. Triplicate repeats were detected and analyzed by TAdivser software (Version 2.0.1.55, Taoyuan, Taiwan).

### 2.11. Color Measurement

Color analysis of fermented mango jam was determined by using a HunterLab colorimeter (Hunter Associates Laboratory, Inc., Reston, VA, USA). For each sample, triplicate measurements were taken in each shell area, and the average of the four samples was recorded. We determined the lightness component L* (range, 0–100), and the chromatic components a* (redness/greenness [+/−]) and b* (yellowness/blueness [+/−]) [25].

### 2.12. Sensory Evaluation

The appearance, odor, texture, flavor, and overall acceptability of each sample were evaluated by panelists. For a trained sensory panel, panelists were trained to isolate each factor and to focus on each independently of the others for evaluation of the property that is of interest to the researcher. There are 20 panelists in this study, including 9 men and 11 women. Assign a rating to each parameter on a 9-point descriptive hedonic scale, in which 9 is reserved for the highest-quality sample [26].

### 2.13. Statistical Analysis

The data were expressed as means ± SD. The statistical analysis was performed by one-way analysis of variance (ANOVA) to compare among groups through Duncan’s multiple-range test (SPSS statistical software package, version 17.0, SPSS, Chicago, IL, USA). Statistic differences with a *p* value < 0.05 were considered statistically significant.

## 3. Results and Discussion

### 3.1. Properties of L. acidophilus BCRC14079-Fermented Mango

**Figure 1:**
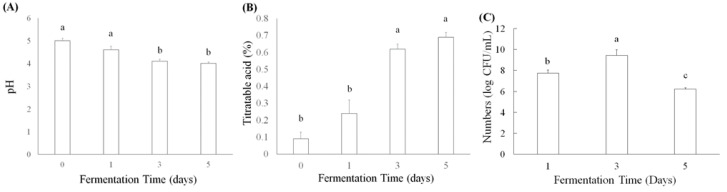
The variations of (**A**) pH, (**B**) titratable acid, and (**C**) microbial numbers in *L. acidophilus* BCRC14079-fermented mango after 1, 3, and 5 days, respectively. Data are shown as means ± SD (*n* = 3). A significant difference was shown by various letters (*p* < 0.05).

*L. acidophilus*, *L. plantarum* subsp. *plantarum*, and *L. paracasei* subsp. *paracasei* have been used to ferment plant products to improve their antioxidation, anti-inflammation, and lipid metabolism, as well as abilities to prevent acute gastric ulcers, antiatherosclerosis, and antiobesity [27,28]. Moreover, some active ingredients, such as γ-aminobutyric acid (GABA) and angiotensin I converting enzyme inhibitor (ACEI), have been found in lactic acid bacteria-fermented products [29]. Titratable acid and pH values were determined in lactic acid bacteria *L. acidophilus* BCRC14079-fermented mango pulp (30%) after 1, 3, and 5 d of fermentation. The pH value decreased significantly (*p* < 0.05), as fermentation progressed from days 3 to 5 (Figure 1A). An increase in titratable acid caused by *L. acidophilus* BCRC14079 produced lactic acid (Figure 1B). Figure 1C shows the results of *L. acidophilus* BCRC14079 growth in mango pulp (30%). The number of *L. acidophilus* BCRC14079 increased after mango pulp had been fermenting for 3 days, which can exceed approximately 10^10^ CFU/mL. However, the number of *L. acidophilus* BCRC14079 was lower on day 5, suggesting that the growth of *L. acidophilus* BCRC14079 in mango pulp as a fermentative material is limited.

### 3.2. Inhibition of the Growth on C. difficile by L. acidophilus BCRC14079-Fermented Mango

The focus of CDI treatment guidelines is to stop continuing using antibiotics and switching to metronidazole and vancomycin [30,31]. However, *C. difficile* develops antibiotic resistance to a number of antibiotics. Therefore, alternative treatment or prevention strategies are needed. The fermented products were centrifuged and divided into *L. acidophilus* BCRC14079-fermented mango supernatant and precipitate. The inhibition of *C. difficile* growth by *L. acidophilus* BCRC14079-fermented mango extracts was evaluated.

**Figure 2:**
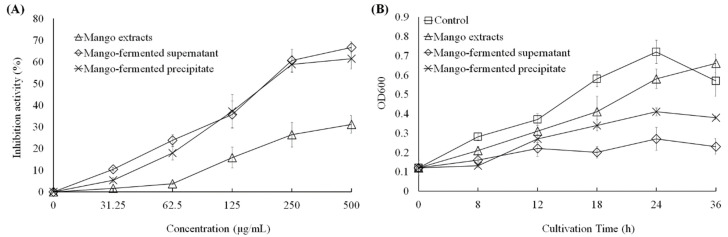
The downregulations of *L. acidophilus* BCRC14079-fermented mango on the growth of *C. difficile* in (**A**) dosage-dependent at 24 h treatment and (**B**) time-dependent manners at 250 μg/mL.

As shown in Figure 2A, the mango extract, *L. acidophilus* BCRC14079-fermented mango supernatant and precipitate could inhibit the growth of *C. difficile* dose dependently (31.25–500 μg/mL). The suppressive effect of *L. acidophilus* BCRC14079-fermented mango supernatant was similar to that of *L. acidophilus* BCRC14079-fermented mango precipitate (24 h treatment). In addition, a dose of 250 μg/mL was used to evaluate the inhibitory effect in *C. difficile* treated with mango extracts, *L. acidophilus* BCRC14079-fermented mango supernatant, and *L. acidophilus* BCRC14079-fermented mango precipitate for 8, 12, 18, 24, and 36 h. As shown in Figure 2B, the ability of *L. acidophilus* BCRC14079-fermented mango supernatant to inhibit *C. difficile* growth was greater than that of the mango extract and *L. acidophilus* BCRC14079-fermented mango precipitate during 18–36 h.

*C. difficile* belongs to Gram-positive bacillus that produces spores. It is an important pathogenic bacterium in adults and children. *C. difficile* was first isolated from the intestinal tract of infants and was clinically important when it became one of the main causes of antibiotic-associated diarrhea [30]. Recurrence is an important hallmark of CDI due to the ability of *C. difficile* to produce resistant spores, partly due to the inability to recover the gut microbiota after antibiotic treatment; importantly, 25% of CDI patients may occur recurrence, and the rate of secondary recurrence can be as high as 40–60% [31,32].

Cytotoxins toxin A and toxin B are cytotoxic proteins, which are the principal virulence factors of *C. difficile*. Toxins A and B of *C. difficile* are secreted and bound to host receptors, subsequently, entered into the cytoplasm of enterocytes [30,31], which causes intestinal inflammation and surface of the epithelial mucosal disruption [33,34]. In a clinical study, probiotic therapy is also considered in CDI treatment and in antibiotics (vancomycin, metronidazole, and fidaxomicin). Therefore, next-generation probiotics or other probiotic species (such as lactic acid bacteria) are promising novel candidates for the development of CDI adjunct therapy. The protective role of *L. casei* LBC80R and *L. acidophilus* CL1285 against CDI has been reported [33]. Moreover, the application of *L. reteri* products on inhibiting *C. difficile* colonization has been evaluated [35]. We found that *L. acidophilus* BCRC14079-fermented mango can inhibit the growth of *C. difficile*.

In a cell model, toxins A and B have been shown to induce cell necrosis and apoptosis in intestinal epithelial cells [36,37]. Therefore, the maintenance of the intestinal barrier is a potential defense against *C. difficile* and reduces the risk of infection. The intestinal mucosa is the first site for the contact between host and pathogen (i.e., virus, bacteria, yeast, protozoa) while infection. Intestinal epithelial cells are able to produce mucin, which can form a mucosal barrier to block tissue represent sits and avoid pathogenic infection.

**Figure 3:**
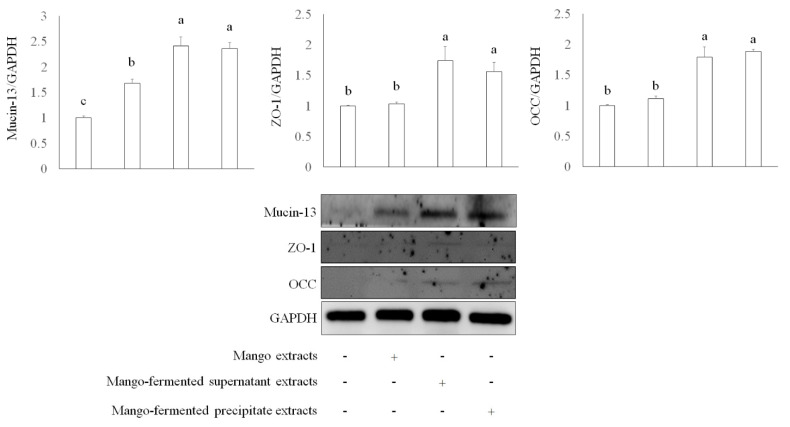
The intestinal protection of *L. acidophilus* BCRC14079-fermented mango on expressions of mucin-13, ZO-1, and OCC in differential Caco-2 cells. Data are shown as means ± SD (*n* = 3). Significant difference was shown by various letters (*p* < 0.05). Symbol + means this sample is added to the group, and symbol − means this sample is not added.

Apart from mucin, epithelial integrity is associated with intestinal tight junction proteins such as zonula occludens-1 (ZO-1) and occludin (OCC). These proteins regulate cellular permeability and maintain intestinal barrier functions [38]. We investigated several tight junction proteins involved in intestinal barrier function, including mucin-13, ZO-1, and OCC in Caco-2 cells treated with *L. acidophilus* BCRC14079-fermented mango. As shown in Figure 3, both *L. acidophilus* BCRC14079-fermented mango supernatant and precipitate (250 μg/mL) treatment significantly increased the expression of mucin-13, ZO-1, and OCC in Caco-2 cells when compared with mango-extract treatment.

**Figure 4:**
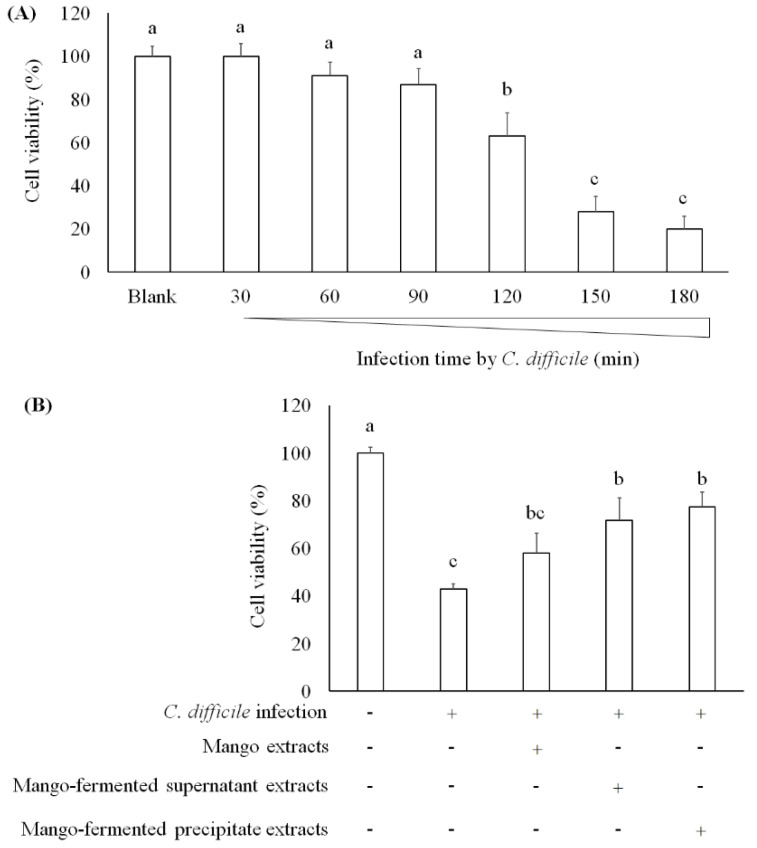
The effect of mango-fermented extracts on the cell viability in Caco-2 cells: (**A**) cell viability was markedly decreased in cells after *C. difficile* infection for 2 h; (**B**) the protection of *L. acidophilus* BCRC14079-fermented mango against *C. difficile* infection in Caco-2 cells. Data are shown as means ± SD (*n* = 3). Significant difference was shown by various letters (*p* < 0.05). Symbol + means this sample is added to the group, and symbol − means this sample is not added.

One study has found that *C. difficile*-derived toxin A causes cell apoptosis and reduction of mucosal integrity in Caco-2 cells [39]. Janvilisri et al. (2010) have tried to induce *C. difficile* infection in Caco-2 cells, and their results showed that the cell viability was decreased after 60 min infection [40]. We investigated the survival of Caco-2 cells treating with *C. difficile* for 30, 60, 90, 120, 150, and 180 min. As shown in Figure 4A, cell viability decreased after *C. difficile* treatment for 120 min. In addition, both *L. acidophilus* BCRC14079-fermented mango supernatant and precipitate extract treatment markedly alleviated the cytotoxic effect of *C. difficile* on Caco-2 cells (Figure 4B). A research study investigates the transcriptomic variation of Caco-2 cells infected with *C. difficile* to understand a framework of host–bacteria interactions. It has been indicated that several biomarkers associated with the epithelial barrier (tight junction proteins) were suppressed by Rho signaling mediation, leading to enterocytes disruption [40,41]. In our study, *L. acidophilus* BCRC14079-fermented mango has the potential to improve the intestinal barrier and attenuate cell death induced by *C. difficile* in Caco-2 cells.

### 3.3. Regulation of L. acidophilus BCRC14079-Fermented Mango on the Growth of F. prausnitzii

**Figure 5:**
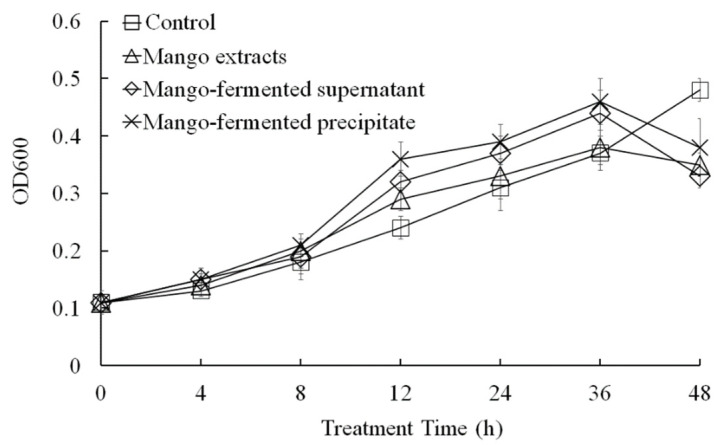
The regulation of *L. acidophilus* BCRC14079-fermented mango on *F. prausnitzii* growth. Data are shown as means ± SD (*n* = 3). Significant difference was shown by various letters (*p* < 0.05).

In this study, we evaluated the effects of *L. acidophilus* BCRC14079-fermented mango on the growth of *F. prausnitzii* (Figure 5). Growth of *F. prausnitzii* was not promoted under culture conditions with the whole mango, *L. acidophilus* BCRC14079-fermented mango supernatant, or *L. acidophilus* BCRC14079-fermented mango precipitate extracts. S supplements of the whole mango, *L. acidophilus* BCRC14079-fermented mango supernatant, or *L. acidophilus* BCRC14079-fermented mango precipitate extracts in YCFA medium (5%) significantly increased growth of *F. prausnitzii* (Figure 5) and production of SCFA (butyrate) (Table 1).

The population of *F. prausnitzii* is potentially regulated by diet and host since it is unable to utilize mucin to be a nutrient, limiting the population within the gut microbiota. However, *F. parausnitzii* would use mucin metabolites after its degradation; hence, mucin may induce the growth of *F. prausnitzii* while this species is under adequate nutritional condition [42]. Several studies have found that the number of *F. parausnitzii* is increased after prebiotics intervention (e.g., fructooligosaccharides, inulin-type fructans, and raffinose). Evidence suggests that some dietary factors may influence the abundance of *F. prausnitzii* [43]. Several factors including fibers, vitamins, and cofactors (biotin, folate, niacin, and thiamine) are essential nutrients to support the growth of *F. prausnitzii* [44]. Mango is a fiber- and vitamins-riched fruit, it may be a good raw material to elevate the growth of *F. prausnitzii*.

*F. prausnitzii* produces short-chain fatty acid butyrate in the colon, which inhibits the growth of *C. difficile*. Moreover, some anti-inflammatory bacteria such as *F. prausnitzii* and lactic acid bacteria support the innate immune response and minimize bacterial burden and negative effects during antibiotic and *C. difficile* exposure [45]. Antioxidants-riched lactic acid bacteria (*L. rhamnosus* R0011) supplements have shown intestinal protection against CDI [46]. Moreover, *F. prausnitzii*-cultured supernatant showed the ability for inhibiting inflammatory cytokines production in Caco-2 cells [47]. SCFAs promote the expression of tight junction proteins in intestinal cells, including occludin and claudin-5, hence reducing the permeability of the blood–brain barrier (BBB) to avoid the entry of pathogenic bacteria or microbial metabolites into blood [48]. For example, when the concentration of propionate is at least 1 μM, the permeability of BBB can be protected [49]. Taken together, we consider that *L. acidophilus* BCRC14079-fermented mango can suppress the growth of *C. difficile*, increase the expression of tight junction proteins, and inhibit *C. difficile* by promoting the growth of *F. prausnitzii* and increasing the level of butyrate. These results show that *L. acidophilus* BCRC14079-fermented mango can be developed as a health product to improve the intestinal microenvironment and gut microbiota, and has the potential to be used in the adjuvant treatment of CDI.

### 3.4. Development of Innovative Probiotics-Fermented Mango Jam

Jam, a kind of medium-moisture food product, is prepared by boiling fruit pulp with sugar, acid, pectin, and/or other substances to achieve a suitable and strong consistency to maintain the texture of the fruit. Jam is usually made by mixing fruit and sugar in a ratio of 45%:55% by weight. Typically, jams are widely consumed for breakfast and incorporated into bakery products and confectionery. With the increasing attention to health and wellness, and the increasing incidence of obesity, metabolic syndrome, and diabetes, the interest in low-calorie foods has also increased [50]. For example, one study produces fiber-fortified fruit jams to enhance the nutrition and texture of jam [51].

HPP, also called high hydrostatic pressure processing, is one kind of cold pasteurization technique by which products with the final package are introduced into a vessel and subjected to 100–700 MPa of isostatic pressure that is transmitted by water. *L. acidophilus* BCRC14079-fermented mango was investigated in this study for developing the fermented jam that contained probiotics. Browning and color loss were found to be higher in the heat-treatment group than the HPP-treatment group under all conditions (Table 2). Hardness is defined as the force required to achieve a certain amount of deformation and is a regular parameter to determine the texture of the jam [52]. In sensory analysis, hardness refers to the force required to press food between the teeth during the first bite [53]. Adhesiveness represents the work required to extract the pressure probe from the sample. In sensory analysis, adhesiveness (viscosity) is the work required to overcome the gravity between the food surface and the food contact surface including teeth, tongue, and palate) [52]. Cohesiveness represents the internal resistance of the food structure, which means the ability to combine product ingredients [54].

**Figure 6:**
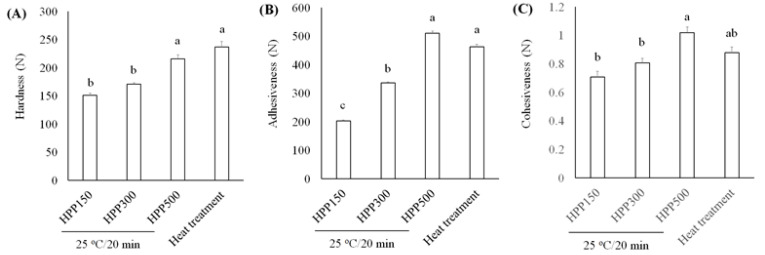
Investigating the textural parameters (**A**) hardness, (**B**) adhesiveness, and (**C**) cohesiveness of *L. acidophilus* BCRC14079-fermented mango jam by HPP and heat treatment. Data are shown as means ± SD (*n* = 3). Significant difference was shown by various letters (*p* < 0.05).

**Figure 7:**
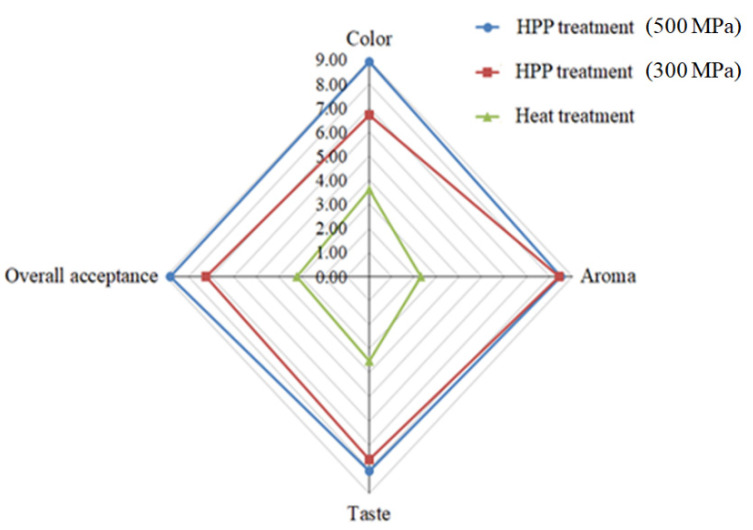
Sensory properties of *L. acidophilus* BCRC14079-fermented mango jam.

We assessed textural characteristics, including hardness, adhesiveness, and cohesiveness (Figure 6). We found that *L. acidophilus* BCRC14079-fermented mango jam had better hardness, adhesiveness, and cohesiveness than that prepared using mango pulp treated with HPP or heat. Moreover, *L. acidophilus* BCRC14079-fermented mango jam with HPP treatment at 500 MPa had the highest sensory quality (aroma, taste, color, and overall acceptance) among all groups (Figure 7). Many foods containing lactic acid bacteria can improve human health and inhibit the growth of pathogens; these probiotics are used to develop innovative foods [55,56]. A novel type of mango jam was produced in this study, which can enhance the population of next-generation probiotics and inhibit the growth of *C. difficile*. This jam was fermented using lactic acid bacteria *L. acidophilus* BCRC14079 and treated with HPP to retain its physicochemical, textural, and rheological properties.

## 4. Conclusions

In this study, *L. acidophilus* BCRC14079-fermented mango products exhibited beneficial effects by promoting the growth of *F. prausnitzii* and protected intestinal cells against *C. difficile* infection via preventing the cell death of Caco-2 cells. This enteroprotective role of *L. acidophilus* BCRC14079-fermented mango products is related to improve the gut barrier function through upregulating tight junction proteins expression and mucin secretion. A novel type of mango jam that was fermented by *L. acidophilus* BCRC14079 and treated with high hydrostatic pressure processing was produced, which had favorable textural characteristics and sensory quality, and had the potential to be developed as an innovative food product for inhibiting the growth of pathogens and improving the human health.

## Figures and Tables

**Table 1:** The production of short-chain fatty acid production in *F. prausnitzii* treated with *L. acidophilus* BCRC14079-fermented mango.

Short-Chain Fatty Acids	Acetic Acid	Propionic Acid	Butyric Acid
	Concentration (μM)		
Control	103.0 ± 13.2 ^c^	9.5 ± 1.5	1477.0 ± 210.6 ^b^
Mango extracts	168.1 ± 18.2 ^b^	11.3 ± 3.1	1892.0 ± 141.7 ^b^
Mango-fermented supernatant extracts	228.3 ± 12.5 ^a^	10.6 ± 2.6	2521.0 ± 115.3 ^a^
Mango-fermented precipitate extracts	241.5 ± 10.6 ^a^	12.8 ± 2.2	2263.0 ± 179.1 ^a^

Data are shown as means ± SD (*n* = 3). Significant difference was shown by various letters (*p* < 0.05).

**Table 2:** The parameters of *L. acidophilus* BCRC14079-fermented mango jam by HPP treatment. L*: lightness component (range, 0–100); a*: chromatic component (redness/greenness [+/−]); b*: chromatic component (yellowness/blueness [+/−]).

Groups	HPP Treatment (MPa)			Heat Treatment
	150	300	500	
*L. acidophilus* BCRC14079-fermented mango solution (%)	53.5	53.5	53.5	53.5
pH	3.05	3.05	3.05	3.05
Total sugars (%)	45	45	45	45
Pectin (%)	1.5	1.5	1.5	1.5
L*	30.76	30.53	31.81	35.49
a*	−3.16	−3.16	−3.14	−2.66
b*	8.14	8.53	8.37	11.24

## Data Availability

Not applicable.
